# Effects of carrageenan, PVP and tumour-bearer serum on immunity induced by excision or mitomycin C-treated tumour cells in mice

**DOI:** 10.1038/bjc.1979.116

**Published:** 1979-06

**Authors:** R. Kearney, R. L. Wu, F. Orr

## Abstract

**Images:**


*Br. J. Cancer* (1979) **39**, 648

### EFFECTS OF CARRAGEENAN, PVP AND TUMOUR-BEARER SERUM ON IMMUNITY INDUCED BY EXCISION OR MITOMYCIN C-TREATED TUMOUR CELLS IN MICE

R. KEARNEY, R. L. WU AND F. ORR

*From the Department of Bacteriology, University of Sydney, Australia*

Received 15 January 1979 Accepted 1 March 1979

**Summary.—Carrageenan (Cg) was tested for its effects on the growth of, and immunity to, 2 methylcholanthrene-induced syngeneic murine fibrosarcomas (H1 and H2). The tumours were found not to share major tumour-specific transplantation antigens. H2 appeared more immunogenic than H1. In contrast to H1, immunity induced by H2 was not affected by Cg, nor was its growth in Cg-treated normal mice augmented.**

**Postoperative i.p. injections of Cg abolished the weak anti-H1 immunity produced by H1 tumour excision. Furthermore, the subsequent growth of the H1 tumour challenge in the Cg-treated *immune* mice was significantly greater than the augmented growth in Cg-treated *normal* mice. The prior administration of the macrophage-stabilizing agent polyvinylpyrrolidone (PVP) to immune mice significantly reduced the augmenting effect of Cg. The growth-promoting effect of Cg on a secondary H1 tumour challenge in mice immunized by tumour excision was abolished by $10^6$ MCT-H1 cells injected s.c. before Cg. In contrast to the immunity induced by tumour excision, Cg did not abolish the immunity induced by the injection of MCT-H1 cells.**

**Passive administration of H1 tumour-bearer serum (TBS) did not enhance the growth of H1 cells in normal mice, nor did TBS abrogate the specific cell-mediated immunity (CMI) induced *in vivo* by MCT-H1 cells. However, TBS administered to Cg-treated, MCT-H1-immune mice abolished tumour immunity.**

**We propose that TBS does not inhibit CMI *in vivo* provided that macrophages remain functional, but may do so when macrophages are rendered defective by anti-macrophage agents or by products of neoplastic cells. Increasing the levels of specific effector cells can over-ride the inhibiting effects of TBS, even when defective macrophages are present.**

TUMOUR-REJECTION IMMUNITY is a complex process in which many components of the lymphoreticular system interact, but the principal effector cells appear to be T lymphocytes and macrophages (Alexander *et al.*, 1972; Evans & Alexander, 1971; Fink, 1976). T lymphocytes separated from tumours have been shown to have specific anti-tumour activity (Gillespie *et al.*, 1977; Plata & Sordat, 1977), and macrophages recovered from tumours appear to be either cytostatic (Evans, 1973; Haskill *et al.*, 1975; Von Loveran & den Otter, 1974) or cytocidal (Russell *et al.*, 1976).

Evidence in support of a role for macrophage-like cells in amplifying lymphocyte killing has been obtained from studies both *in vitro* (Kearney *et al.*, 1975) and *in vivo* (Simes *et al.*, 1975). More direct evidence for the participation of macrophages in tumour rejection has been obtained by attempts to ablate macrophages *in vivo* by a variety of agents including carrageenan, silica or trypan blue (Zarling & Trevethia, 1973; Keller,

Correspondence: R. Kearney, Department of Bacteriology, University of Sydney, Sydney, N.S.W. 2006, Australia.

1976; Kripke *et al.*, 1977; Thomson & Fowler, 1977; Nelson & Nelson, 1978). Treatment by these agents, known to diminish the functional capacity of macrophages *in vitro*, led to markedly augmented growth of many tumours in normal animals.

The aim of the present investigation was to establish whether 2 chemically-induced syngeneic tumours produce specific immunity after either the administration of mitomycin-C-treated tumour cells (Benjamini *et al.*, 1977) or the surgical removal of tumour isografts, and to determine whether the administration of carrageenan altered the immunity induced by either of the above methods.

### MATERIALS AND METHODS

*Animals.*—Male mice (8–12 weeks old) of the highly inbred CBA/H/WEH1 strain were used. Their origin and maintenance have been discussed previously (Basten *et al.*, 1974).

*Tumours.*—Two syngeneic methylcholanthrene-induced fibrosarcomas designated H1 and H2 were used. Tumour-cell suspensions were prepared from freshly excised tumours by gently disaggregating tumour fragments in 5–10 ml of 0·1% pronase in Dulbecco-modified Eagles' medium (DME) as described previously (Kearney & Nelson, 1973). The cell suspension obtained was washed ×3 in DME containing 15% foetal calf serum (FCS). Cells were finally suspended in serum-free medium and the the viability determined by trypan-blue exclusion. Viable tumour-cell suspensions in serum-free medium were adjusted to the appropriate concentration indicated for particular experiments. Viable tumour cells were injected s.c. in a volume of 0·2 ml along the midline of the abdominal wall. At daily intervals, usually after the 4th day, the smallest and largest diameters of the tumours were measured (to the nearest 0·1 mm) by a Schnelltaster dial gauge (Kearney & Nelson, 1973) or by vernier calipers. The tumour diameter was expressed as the average of both diameters, and corrected for average thickness of the uninjected mouse skin-fold of the abdominal wall. The mean tumour diameters are re-recorded with their standard errors.

*Excision and rechallenge assays of tumour immunity.*—Mice were immunized with living tumour inoculated s.c. in the flank, followed by tumour excision. The faster-growing H1 tumour was inoculated in a dose of $10^6$ cells, which developed 10–15mm sarcomas within 10–12 days, when they were excised under ether anaesthesia. The slower-growing H2 tumour, in a dose of $10^7$ cells, grew to about the same size within 12–14 days, when they were similarly excised. Six weeks after surgical excision, these mice and control mice were challenged s.c. along the midline of the abdominal wall with the appropriate challenge dose as indicated in the particular experiments.

*Preparation of mitomycin-C-treated tumour cells (MCT cells).*—Tumour cells obtained by incubation with 0·1% pronase in DME medium were washed ×3 in DME medium with 20% FCS. Mitomycin C (MC) (Kyowa Hakko Kygyo Co. Ltd, Japan) was dissolved in DME with 20% FCS and then added to the tumour cells at a concentration of 30 $\mu$g/$10^6$ H1 tumour cells/ml medium. The cells were incubated at 37°C for 35 min, and then washed ×3 in medium alone to remove free MC and FCS. Viability of total nucleated cells treated in this way usually exceeded 95%. Recurrence of tumour growth of MCT cells was not observed in the present experiments. A single dose of $10^6$ MCT cells was injected s.c. in the flank to induce specific immunity (Wu & Kearney, submitted for publication) and these mice were challenged 14 days later.

*Carrageenan* (Cg).—Lambda carrageenan (Marine Colloids Inc., Springfield, New Jersey) was dissolved in boiling saline at a concentration of 0·5 mg/0·2 ml, and then stored at −20°C until required. The amount of Cg administered varied according to the particular experiment but in general, animals were given 0·5 mg of Cg injected i.p. on each of 3 days before the test procedure, *i.e.*, before inoculation of MCT tumour cells, or before subsequent challenge-inoculation with viable tumour cells. On the day of the experiment, a final dose of 0·5 mg was administered about 1 h before the antigen. Thus, a total dose of 2 mg was administered in 4 fractions.

*Polyvinylpyrrolidone* (PVP).—PVP (May and Baker Ltd, Dagenham, England; Lot 49614), mol. wt 30,000–40,000, was dissolved in saline at a concentration of 8 mg/ml. One day before the first dose of Cg, mice were injected i.p. with 0·5 ml PVP. On the following day, 1 h before mice received Cg, 0·25 ml PVP was injected both i.p. and s.c. Thus a total of 20 mg PVP was injected into each mouse over a 5-day period before tumour challenge.

### RESULTS

Preliminary experiments established that the threshold doses of $0{\cdot}5\times10^5$ for the H1 tumour and $5\times10^5$ for the H2 tumour would develop into palpable tumours in all mice within 2 weeks, and were therefore used as the standard challenge inocula to detect weak immunity in the *in vivo* assays.

### *Magnitude of the immune response to the H1 tumour after tumour excision*

The strength of the resistance acquired by H1 tumour excision was assessed by injecting mice (in groups of 8–12) with $0{\cdot}5\times10^5$, $1\times10^5$ and $5\times10^5$ viable H1 cells.

Fig. 1 shows that tumour resistance 6 weeks after excision is weak, since a dose of only $0{\cdot}5\times10^5$ cells, but not of $5\times10^5$ cells, was completely rejected. The growth rate in the latter mice was less initially than that of the control mice, suggesting that the growth of some tumour cells was arrested. Mice challenged with an intermediate dose of $10^5$ cells showed variable resistance.

FIG. 1.—Effect of dose of H1 tumour challenge inoculated s.c. into the abdominal wall of normal CBA mice, and mice immunized by removal of syngeneic H1 tumour isograft 6 weeks before. Normal mice challenged with: $0{\cdot}5\times10^5$, ●——●; $1\times10^5$, ○——○; $5\times10^5$, ▲——▲. Immune mice challenged with: $0{\cdot}5\times10^5$ cells, ● - - - ●; $1\times10^5$ cells, (○); $5\times10^5$ cells, ▲ - - - ▲. Each point on the lines represents the mean values for 8–12 mice per group.

FIG. 2.—The effect of carrageenan (Cg) on the growth of $0{\cdot}5\times10^5$ syngeneic H1 tumour cells inoculated s.c. into the abdominal wall of normal CBA mice, and mice immunized by excision of H1 tumour isografts 6 weeks before.

Growth of H1 cells in: 8 normal mice, ●——●; 8 Cg-treated normal mice, ○——○; 11 immune mice, ■——■; 10 Cg-treated immune mice, ○ - - - ○.

*Effect of carrageenan on the tumour resistance of mice immunized by the excision of H*1 *tumour isografts*

To assess the effect of Cg on tumour resistance acquired by tumour excision, immune mice (12–14 per group) were injected i.p. with a total of 2 mg Cg given in 4 daily 0·5 mg doses during the 6th week after H1 tumour excision. The last dose of Cg was given 1 h before a tumour challenge of $0{\cdot}5 \times 10^5$ H1 cells s.c.

Fig. 2 shows that Cg abolished the immunity to the H1 tumour inoculum, and that the subsequent tumour growth was significantly greater (from Day 9) than the augmented growth in Cg-treated *normal* mice.

*Effect of carrageenan on the growth of H*1 *and H*2 *tumours in mice immunized by MCT-H*2 *inoculation*

Eight mice in each of 4 groups were injected s.c. in the flank with $10^6$ MCT-H2 cells. Mice in 2 of the MCT-H2 treated groups were injected i.p. with 2 mg Cg in 4 daily 0·5mg doses before tumour challenge 14 days after MCT inoculation. Control mice with or without Cg were included. Mice were challenged with either $0{\cdot}5 \times 10^5$ H1 or $5 \times 10^5$ H2 tumour cells injected s.c.

Fig. 3 shows that the immunity induced by MCT-H2 injection led to the rejection of $5 \times 10^5$ H2 tumour cells but not of $0{\cdot}5 \times 10^5$ H1 tumour cells. Cg did not affect the expression of MCT-H2 resistance to the H2 tumour, nor did Cg augment the growth of H2 tumours in normal mice. Cg did, however, augment the growth of H1 tumour cells inoculated into MCT-H2-immune mice. No evidence was found, therefore, that H1 and H2 tumours shared major tumour-specific transplantation antigens.

*Effect of carrageenan on the growth on H*1 *and H*2 *tumours in mice immunized by excision of H*2 *tumour isografts*

Concurrent with the previous experiments, 8 mice, in each of 4 groups, immunized by excision of H2 tumour

FIG. 3.—Effect of carrageenan (Cg) on the growth of syngeneic H1 and H2 tumours injected s.c. into the abdominal wall of normal CBA mice, and mice immunized by $10^6$ mitomycin-C-treated H2 tumour cells (MCT-H2) 14 days before. Cg (2 mg) was administered i.p., after MCT-H2 treatment, over a 3-day period before secondary tumour challenge.

Growth of $0{\cdot}5 \times 10^5$ H1 cells in: normal mice, ●——●; Cg-treated normal mice, ○——○; MCT-H2-immune mice, ▲——▲; Cg-treated MCT-H2-immune mice, △——△.

Growth of $5 \times 10^5$ H2 cells in: normal mice, ■ - - - - ■; Cg-treated normal mice, □ - - - - □; MCT-H2-immune mice, ■——■; Cg-treated MCT-H2-immune mice, □——□.

Each point represents the mean of 8 mice.

isografts, were challenged 6 weeks later with either $0{\cdot}5 \times 10^5$ H1 or $5 \times 10^5$ H2 tumour cells. Immune mice in 2 of the groups were pretreated with Cg as already described. Control mice of similar numbers were included.

Fig. 4 shows that excision of the H2 tumour isograft led to total resistance to the H2 tumour, but was ineffective against the H1 tumour. In contrast to the augmenting effect of Cg on H1 tumour growth in mice immunized by excision of the H1 tumour, Cg did not abolish the specific resistance acquired by H2 tumour excision, thus indicating other differences

FIG. 4.—Effect of carrageenan (Cg) on the growth of syngeneic H1 and H2 tumours injected s.c. into the abdominal wall of normal CBA mice, and mice immunized by excision of H2 tumour isografts 6 weeks before. Cg (2 mg) was administered i.p. over a 3-day period before secondary challenge.

Growth of $0{\cdot}5 \times 10^5$ H1 cells in: normal mice, ●——●; Cg-treated normal mice, ○——○; H2-immune mice, ▲——▲; Cg-treated H2-immune mice, △——△.

Growth of $5 \times 10^5$ H2 cells in: normal mice, □- - - -□; H2 immune mice, ■- - -■; Cg-treated H2 immune mice. ○- - -○.

Each point on the graph represents the mean of 8 mice.

in the properties of the non-cross-reacting H1 and H2 tumours.

### *Abolition of carrageenan-induced augmentation of growth of H1 tumour in mice immune by pretreatment with MCT-H1 cells*

Previous studies (Wu & Kearney, submitted for publication) had shown that mice injected s.c. with a single dose of $10^6$ MCT-H1 cells developed specific immunity which, after 7 days, resisted a challenge inoculum of $0{\cdot}5 \times 10^5$ H1 cells. Only partial resistance, however, was obtained against challenge doses greater than 0·5 $\times 10^5$ H1 cells in such mice.

The following experiments were designed to test whether a single inoculation of $10^6$ MCT-H1 cells, before the administration of Cg to mice, prevented the augmented tumour growth found when Cg was administered to mice immunized by tumour excision. Sixteen mice, in each of four groups, were immunized by excising H1 isografts as described. Four weeks later, immune mice in 2 of the groups were injected s.c. in the flank with $10^6$ MCT-H1 cells. During the 6th week, immune mice in one of the MCT-treated groups were injected i.p. with 2 mg Cg as before. Of the

FIG. 5.—Effect of the administration of $10^6$ mitomycin-C-treated H1 tumour cells (MCT-H1) on the enhancing effect of Cg on H1 tumour challange in CBA mice immunized by the excision of syngeneic H1 tumour isografts 6 weeks before. Cg (2 mg) was administered i.p. over a 3-day period before secondary tumour challenge. MCT-H1 cells were injected s.c. 14 days before secondary tumour challenge.

Growth of $0{\cdot}5 \times 10^5$ H1 cells in: normal mice, ●——●; Cg-treated normal mice, ○——○; tumour-excised immune mice, ■——■; Cg-treated, tumour-excised immune mice, ○- - -○; MCT-H1-injected mice, ▲——▲; Cg-treated, MCT-H1 injected mice, △——△; Cg-treated, MCT-H1 injected, tumour-excised immune mice, □——□.

Tumour-excised immune mice, pretreated with MCT-H1 cells, all resisted growth (not shown) of the challenge inoculum. Each point represents the mean of 8–12 mice.

2 remaining groups of immune mice, one was given Cg while the other was left untreated. Eight normal mice in each of the control groups were similarly treated with MCT-H1 cells or Cg, or both. In the 6th week, all mice, including 8 untreated mice, were challenged with $0{\cdot}5 \times 10^5$ H1 cells injected s.c. soon after the last dose of Cg.

Fig. 5 shows that although Cg abolished the resistance acquired by tumour excision, it did not inhibit the immunity induced by a single injection of $10^6$ MCT-H1 cells. Furthermore, results show that the augmenting effect of Cg in mice immunized by tumour excision can be prevented by a single s.c. injection of $10^6$ MCT-H1 cells 14 days before tumour challenge. Mice immunized by tumour excision and treated with MCT-H1 cells retain their immunity (results not shown).

*Effect of polyvinylpyrrolidone (PVP) on the tumour-augmenting property of Cg in normal and immune mice*

Mice, in groups of 6–8, immunized by H1 tumour excision, were treated with either PVP, Cg or both before secondary H1 challenge 8 weeks after tumour excision. Table I shows the mean diameters of the tumours in mice of both test and control groups 19 days after challenge with $0{\cdot}5 \times 10^5$ H1 cells. PVP significantly reduced the tumour-promoting effect of Cg in the groups of immune and normal mice. Fig. 6 illustrates the relative sizes of representative tumours from each of the groups.

*Effect of serum from animals with progressing tumours on immunity of animals immunized with MCT cells*

Although MCT-H1 cells induce an effective, cell-mediated anti-tumour resistance, this response could not be detected when MCT cells were injected into animals with established tumours (Wu & Kearney, submitted for publication). One possibility is that animals with growing tumours have serum factors which can prevent the expression of cell-mediated cytotoxic effects on tumour cells. This effect has been detected by *in vitro* assays (Hellström & Hellström, 1974) but their relevance *in vivo* has been questioned.

The following experiments are based on the observation that the response to MCT cells appears to be only cell-mediated (Benjamini *et al.*, 1977; Wu & Kearney, submitted for publication) and that therefore any serum factor present in tumour-bearing mice which interferes with cell-mediated mechanisms should be easily detected. Tumour-bearer serum (TBS) was obtained by bleeding mice 4, 5, 6 and 7 days after s.c. inoculation of $2 \times 10^6$ live H1 cells in the flank. Serum samples were pooled from 20–30 donor animals and stored at −20°C. Normal mouse serum

TABLE I.—*Inhibition by polyvinylpyrrolidone (PVP) of the growth-augmenting effect of carrageenan (Cg) on the H1 tumour in normal and immune mice*

| Code | H1 tumour isograft and excision (Exc) | PVP treatment; 4 mg daily, 5 days before challenge | Cg treatment; 0·5 mg daily, 4 days before challenge | Diam. of tumour challenge in site at Day 19 Mean ± s.e. (Units 0·1 mm) |
|---|---|---|---|---|
| N/H1 | − | − | − | 125 ± 7 |
| N/PVP/H1 | − | + | − | 98 ± 8 |
| N/Cg/H1 | − | − | + | 156 ± 10 |
| N/PVP/Cg/H1 | − | + | + | 135 ± 9 |
| Exc/H1 | + | − | − | 0 |
| Exc/PVP/H1 | + | + | − | 0 |
| Exc/Cg/H1 | + | − | + | 181 ± 15 |
| Exc/PVP/Cg/H1 | + | + | + | 70 ± 4 |

Mice challenged s.c. with $0{\cdot}5 \times 10^5$ H1 tumour cells 8 weeks after tumour excision.

FIG. 6.—Effect of PVP growth of H1 tumours in normal and immune mice treated or not with Cg after tumour excision. Photo illustrates the size of 2 representative tumours in each group shown in Table I 19 days after secondary challenge inoculation with $0{\cdot}5 \times 10^5$ H1 cells. Explanation of code in Table I.

(NMS) was pooled from 20–30 donor mice. In this experiment MCT-H1-immunized mice were injected i.p. with 0·9 ml of TBS or 0·9 ml of NMS on the 14th day after immunization. Unimmunized age-matched controls received the same treatment. One hour after receiving serum, all animals were injected s.c. with $0{\cdot}5 \times 10^5$ H1 cells along the midline of the abdominal wall. Results are shown in Table II and Fig. 7, where it is seen that TBS appeared to have no effect on the growth of the tumour in normal mice, and no effect on the ability of immunized mice to express resistance.

*Effect of tumour-bearer serum on immunity in carrageenan-treated animals immunized with MCT-H1 cells*

Mice were immunized with MCT-H1 cells as in the previous experiment. Beginning 10 days after immunization, half

TABLE II.—*Effect of normal and tumour-bearer serum on immunity induced by injection of mitomycin-C-treated H1 tumour cells in mice*

| Immunization | Serum 0·9 ml i.p. | Tumour incidence at Day 17 |
|---|---|---|
| — | — | 8/8 |
| — | NMS | 7/7 |
| — | TBS | 8/8 |
| + | — | 0/5 |
| + | NMS | 3/6 |
| + | TBS | 0/7 |

NMS, normal mouse serum; TBS, tumour-bearer serum. $10^6$ MCT-H1 cells injected s.c. into the flank to immunize. Donor TBS was obtained by bleeding mice 4, 5, 6, and 7 days after inoculation of $2\times10^6$ live H1 cells. Recipient mice were injected i.p. 14 days after sensitization with MCT-H1 cells, and 1 h before challenge with $0{\cdot}5\times10^5$ H1 cells.

FIG. 7.—Effect on H1 tumour growth of 0·9 ml tumour-bearer serum injected i.p. ●——●, $0{\cdot}5\times10^5$ H1 tumour alone, s.c.; ▲- - - -▲, $0{\cdot}5\times10^5$ H1 tumour cells + normal mouse serum; ○ - - - ○, $0{\cdot}5\times10^5$ H1 tumour cells + tumour-bearer serum.

the mice and the same number of age-matched unimmunized controls began Cg treatment. On the 14th day after immunization, animals received 1·0 ml of TBS (collected 5, 6, 7 and 8 days after a challenge of $3\times10^6$ H1 cells) or NMS by i.p. injection. One hour later the animals were challenged with $0{\cdot}5\times10^5$ H1 cells s.c. as before. Results recorded in Table III confirm the previous observation, and show that the immunity of mice given MCT-H1 cells could be abrogated by pre-

TABLE III.—*Effect of normal and tumour-bearer serum in normal and immune mice treated with or without carrageenan (Cg)*

| Immunization[1] | Cg | Serum 1·0 ml i.p. | Tumour incidence at Day 18 |
|---|---|---|---|
| — | — | — | 8/8 |
| — | + | — | 8/8 |
| — | — | NMS[1] | 7/7 |
| — | + | NMS | 7/7 |
| — | — | TBS[1] | 8/8 |
| — | + | TBS | 8/8 |
| + | — | — | 1/7 |
| + | + | — | 0/7 |
| + | — | NMS | 2/7 |
| + | + | NMS | 1/6 |
| + | — | TBS | 1/6 |
| + | + | TBS | 6/7 |

[1] As in Table II (q.v.). Lambda carrageenan; 2 mg injected i.p. between Days 10 and 14 after MCT-H1 inoculation. TBS collected from donor mice 5, 6, 7, and 8 days after inoculation with $3\times10^6$ H1 cells. Recipient mice were injected i.p. 14 days after MCT-H1 inoculation, and 1 h before challenge with $0{\cdot}5\times10^5$ H1 tumour cells.

treatment with Cg *together* with administration of TBS, although either of these treatments alone had no effect (Table III).

In Tables II and III, the tumours which occurred in immune mice treated with NMS were significantly smaller than those in normal mice treated with NMS.

### DISCUSSION

Cg has many biological effects (Di Rosa, 1972) including inhibition of blood coagulation (Anderson & Duncan, 1965) and the complement system (Davies, 1965). Cg also has the ability to interfere with antibody reactions *in vivo* (Ishizaka *et al.*, 1977). In addition, the substance is toxic for macrophages *in vitro* (Catanzaro *et al.*, 1971) and *in vivo* (Nelson & Nelson, 1978). Its augmenting effect upon syngeneic tumours has been reported by several workers (Keller, 1976; Thomson & Fowler, 1977; Nelson & Nelson, 1978). Keller (1976) suggested that the augmented tumour growth could be explained by a diminished period of macrophage function leading to successful tumour implantation and initiation of progressive growth. In support of this interpretation, Keller (1976) found that the augmenting effects of Cg (and silica) on tumour growth were reversed by the macrophage-stabilizing agent poly-2-vinylpyridine N-oxide (PVNO)—a substance known to reverse the immunosuppressive effect of silica and Cg (Rios & Simmons, 1972). However, Keller (1976) observed that tumour growth could also be increased by a growth-promoting agent released from damaged macrophages.

In the present experiments, Cg greatly increased the growth of the low-immunogenic H1 tumour in mice immunized by tumour excision and then treated with Cg before secondary challenge. In direct contrast, however, Cg had no effect on the same tumour when inoculated into mice immunized by MCT-H1 cells. This difference must be due to the differences in the type of immunity induced by tumour excision and MCT inoculation. Evidence by Benjamini *et al.* (1977) and Wu & Kearney (submitted for publication) revealed that the development of MCT-induced immunity was paralleled by the development of specific cell-mediated cytotoxicity without the formation of detectable anti-tumour antibodies. On the other hand, specific immunity acquired by *tumour excision* is mediated by both cytotoxic cells (Belehradek *et al.*, 1972) and antibody (Pilch & Riggins, 1966). By using a modification of the method described by Brown *et al.* (1977), sera from H1 tumour-bearing mice and from mice immunized by tumour excision were found to possess specific antibody to the H1 tumour (Kearney, to be published). However, similar antibody activity could not be found in sera of mice immunized by MCT-H1 cells. Therefore, in addition to the magnitude of the immune responses, a major factor which could also determine whether tumour growth is enhanced or not in immune mice pretreated with Cg is whether antibody accompanies the cell-mediated response.

The *in vitro* cell-mediated response to the H1 tumour is feeble, and depends upon the amplification of the weak cytotoxic effect of the T-cell component by macrophage-like cells (Kearney *et al.*, 1975; Nelson *et al.*, 1978). The present experiments show that the immunity acquired *in vivo* by either surgical removal of the H1 tumour or MCT treatment is also weak. The effect of Cg on macrophages could account for the even lower resistance found in mice whose primary isograft had been removed. The macrophage-stabilizing properties of PVP (Hochschild, 1973) largely reversed the augmenting effect of Cg in mice immunized by H1-tumour excision. The situation is clearly different in MCT-immunized mice, in which the immunity is not abrogated by Cg. Possibly the numbers of specific effector T cells in Cg-treated MCT-immune mice were able to control the growth of a challenge tumour inoculum in the *absence of antibody* without the presence of functional macrophages.

The loss of immunity in Cg-treated mice immunized by tumour excision may have been due to the inhibition of cytotoxic effector cells by a serum factor, presumably antibody, in the *absence* of functional macrophages.

Cg does not abolish pre-existing humoral immunity to sheep erythrocytes, but the secondary response to sheep erythrocytes in Cg-treated mice is delayed (Kearney & Orr, unpublished). A similar delay in the secondary antibody response to the H1 tumour challenge could account for the delay in the enhancement phenomenon in Cg-treated mice immunized by tumour excision (see Figs. 2 & 5). The abolition of Cg-induced augmentation by prior inoculation of MCT cells in mice immunized by tumour excision may be attributed to an increase in the level of effector cells akin to a booster-like effect seen when irradiated cells are administered after surgical excision (Le François *et al.*, 1971; Belehradek *et al.*, 1972). Such an increase in the level of specific cytotoxic cells may override the inhibiting effect of antibody, and also compensates for a loss of accessory macrophages rendered non-functional by Cg. This view is consistent with the observation of

Simes *et al.* (1975), in which small numbers of lymphocytes were ineffective in killing similar tumour cells *in vivo* without accessory radio-sensitive marrow-derived cells (presumably macrophages), whilst larger numbers were effective alone.

Taken together, these findings may help to explain the failure of Cg to augment the growth of the H2 tumour in normal mice, and the failure of Cg to abolish resistance in immune mice. Such an escape from the effects of Cg may depend upon the H2 tumour being more immunogenic and thus inducing greater immune response which is not completely dependent upon the presence of macrophages for its expression.

In view of previous observations and those reported in the present study, we wish to set forth an hypothesis to explain certain features of tumour growth in Cg-treated normal and immune mice.

We propose that tumour-bearer serum does not abrogate cell-mediated immunity to tumours *in vivo* when macrophages are functional. Cg, a known inhibitor of macrophage function, will augment the growth of weakly immunogenic tumours. In situations where the cell-mediated immunity is weak, the presence of defective macrophages does not negate the cytotoxic T-cell function, provided antibody is absent, as in MCT-induced immunity. However, specific antibody and defective macrophages, if present when specific cell-mediated cytotoxicity is weak, will abolish cellular resistance, as seen in mice with feeble immunity induced by tumour-excision and subsequently treated with Cg. In contrast, the blocking effect of immune serum, in the presence of defective macrophages, is abolished when the population of specific effector cells is increased, as seen when mice, immunized by tumour excision, are boosted by MCT cells before Cg treatment. This explains why tumours which induce strong cell-mediated immunity are less likely to be affected by anti-macrophage agents. Weak immunogenicity would seem to confer a significant biological advantage to tumour cells.

This work was supported by grants from the University of Sydney Cancer Research Committee, New South Wales State Cancer Council and the University of Sydney Medical Research Committee.

The assistance of Mrs Barbara Taylor in part of this work is gratefully acknowledged.

### REFERENCES

ALEXANDER, P., EVANS, R. & GRANT, C. K. (1972) The interplay of lymphoid cells and macrophages in tumour immunity. *Ann. Inst. Pasteur*, **122**, 645.

ANDERSON, W. & DUNCAN, J. G. (1965) The anticoagulant activity of carrageenan. *J. Pharm. Pharmacol.*, **17**, 647.

BASTEN, A., MILLER, J. F. A. P., SPRENT, J. & CHEERS, C. (1974) Cell-to-cell interaction in the immune response. X. T-cell dependent suppression in tolerant mice. *J. Exp. Med.*, **140**, 199.

BELEHRADEK, J., BARSKI, G. & THONIER, J. (1972) Evolution of cell-mediated antitumour immunity in mice bearing a syngeneic chemically induced tumour. Influence of tumour growth, surgical removal and treatment with irradiated tumour cells. *Int. J. Cancer*, **9**, 461.

BENJAMINI, E., FONG, S., ERICKSON, C., LEUNG, C. Y., RENNICK, D. & SCIBIENSKI, R. J. (1977) Immunity to lymphoid tumours induced in syngeneic mice by immunization with mitomycin C-treated cells. *J. Immunol.*, **118**, 685.

BROWN, J. P., KLITZMAN, J. M. & HELLSTRÖM, K. E. (1977) A microassay for antibody binding to tumor cell surface antigens using $^{125}$I-labelled protein A from *Staphylococcus aureus*. *J. Immunol. Methods*, **15**, 57.

CATANZARO, P. J., SCHWARTZ, H. J. & GRAHAM, R. C. (1971) Spectrum and possible mechanism of carrageenan cytotoxicity. *Am. J. Pathol.*, **64**, 387.

DAVIES, G. E. (1965) Inhibition of complement by carrageenan: Mode of action, effect of allergic reactions and on complement of various species. *Immunology*, **8**, 291.

DI ROSA, M. (1972) Biological properties of carrageenan. *J. Pharm. Pharmacol.*, **24**, 89.

EVANS, R. (1973) Macrophages and the tumour-bearing host. *Br. J. Cancer*, **28** (Suppl 1), 19.

EVANS, R. & ALEXANDER, P. (1971) Rendering macrophages specifically cytotoxic by a factor released from immune lymphoid cells. *Transplantation*, **12**, 227.

FINK, M. A. (1976) *The Macrophage in Neoplasia.* New York: Academic Press.

GILLESPIE, G. Y., HENSEN, C. B., HOSKINS, R. G. & RUSSELL, S. W. (1977) Inflammatory cells in solid murine neoplasms. IV Cytolytic T-lymphoctyes isolated from regressing or progressive Moloney sarcomas. *J. Immunol.*, **119**, 564.

HASKILL, J. S., PROCTOR, J. W. & YAMAMURA, Y. (1975) Host responses within solid tumours. I. Monocytic effector cells within rat sarcomas. *J. Natl Cancer Inst.*, **54**, 387.

HELLSTRÖM, K. E. & HELLSTRÖM, I. (1974) Lymphocyte mediated cytotoxicity and blocking serum activity to tumour antigens. *Adv. Immunol.*, **18**, 209.

HOCHSCHILD, R. (1973) Effects of various additives on *in vitro* survival time of mouse macrophages. *J. Gerontol.*, **28**, 447.

ISHIZAKA, S., OTANI, S. & MORISAWA, S. (1977) Effects of carrageenan on immune responses. I.

Studies on the macrophage dependency of various antigens after treatment with carrageenan. *J. Immunol.*, **118,** 1213.

KEARNEY, R. & NELSON, D. S. (1973) Concomitant immunity to syngeneic methylcholanthrene-induced tumours in mice. Occurrence and specificity of concomitant immunity. *Aust. J. Exp. Biol. Med. Sci.*, **51,** 723.

KEARNEY, R., BASTEN, A. & NELSON, D. S. (1975) Cellular basis for the immune response to methylcholanthrene-induced tumours of mice. Heterogeneity of effector cells. *Int. J. Cancer*, **15,** 438.

KELLER, R. (1976) Promotion of tumour growth *in vivo* by anti-macrophage agents. *J. Natl Cancer Inst.*, **57,** 1355.

KRIPKE, M., NORBURY, K. C., GRUYS, E. & HIBBS, J. B. (1977) Effects of trypan blue treatment on the immune response of mice. *Infect. Immun.*, **17,** 121.

LE FRANÇOIS, D., YOUN, J. K., BELEHRADEK, J. & BARSKI, G. (1971) Evolution of cell-mediated immunity in mice bearing tumours produced by a mammary carcinoma cell line. Influence of growth, surgical removal and treatment with irradiated tumour cells. *J. Natl Cancer Inst.*, **46,** 981.

NELSON, D. S., HOPPER, K. E., BLANDEN, R. V., GARDNER, I. D. & KEARNEY, R. (1978) Failure of immunogeneic tumours to elicit cytolytic T-cells in syngeneic hosts. *Cancer Lett.*, **5,** 61.

NELSON, M. & NELSON, D. S. (1978) Macrophages and resistance to tumours. Influence of agents affecting macrophages and delayed-type hypersensitivity on resistance to tumours inducing concomitant immunity. *Aust. J. Exp. Biol. Med. Sci.*, **56,** 211.

PILCH, Y. H. & RIGGINS, R. S. (1966) Antibodies to spontaneous and methylcholanthrene-induced tumours in inbred mice. *Cancer Res.*, **26,** 871.

PLATA, F. & SORDAT, B. (1977) Further characterization of murine sarcoma virus (MSV)—Immunocytolytic T-lymphocytes *in vivo*. *Int. J. Cancer*, **19,** 205.

RIOS, A. & SIMMONS, R. L. (1972) Poly-vinyl-pyridine-N-oxide reverses the immunosuppressive effect of silica and carrageenan. *Transplantation*, **13,** 343.

RUSSELL, S. W., DOE, W. F., HOSKINS, R. G. & COCHRANE, C. G. (1976) Inflammatory cells in solid murine neoplasms. I. Tumour disaggregation and identification of constituent inflammatory cells. *Int. J. Cancer*, **18,** 322.

SIMES, R. J., KEARNEY, R. & NELSON, D. S. (1975) Role of a noncommitted accessory cell in the *in vivo* suppression of a syngeneic tumour by immune lymphocytes. *Immunology*, **29,** 343.

THOMSON, A. W. & FOWLER, E. W. (1977) Potentiation of tumour growth of carrageenan. *Transplantation*, **24,** 397.

VON LOVEREN, H. & DEN OTTER, W. (1974) Macrophages in solid tumours. I. Immunologically specific effector cells. *J. Natl Cancer Inst.*, **53,** 1057.

ZARLING, J. M. & TEVETHIA, S. S. (1973) Transplantation immunity to Simian Virus 40-transformed cells in tumour-bearing mice. II. Evidence for macrophage participation at the effector cell level of tumour cell rejection. *J. Natl Cancer Inst.*, **50,** 149.


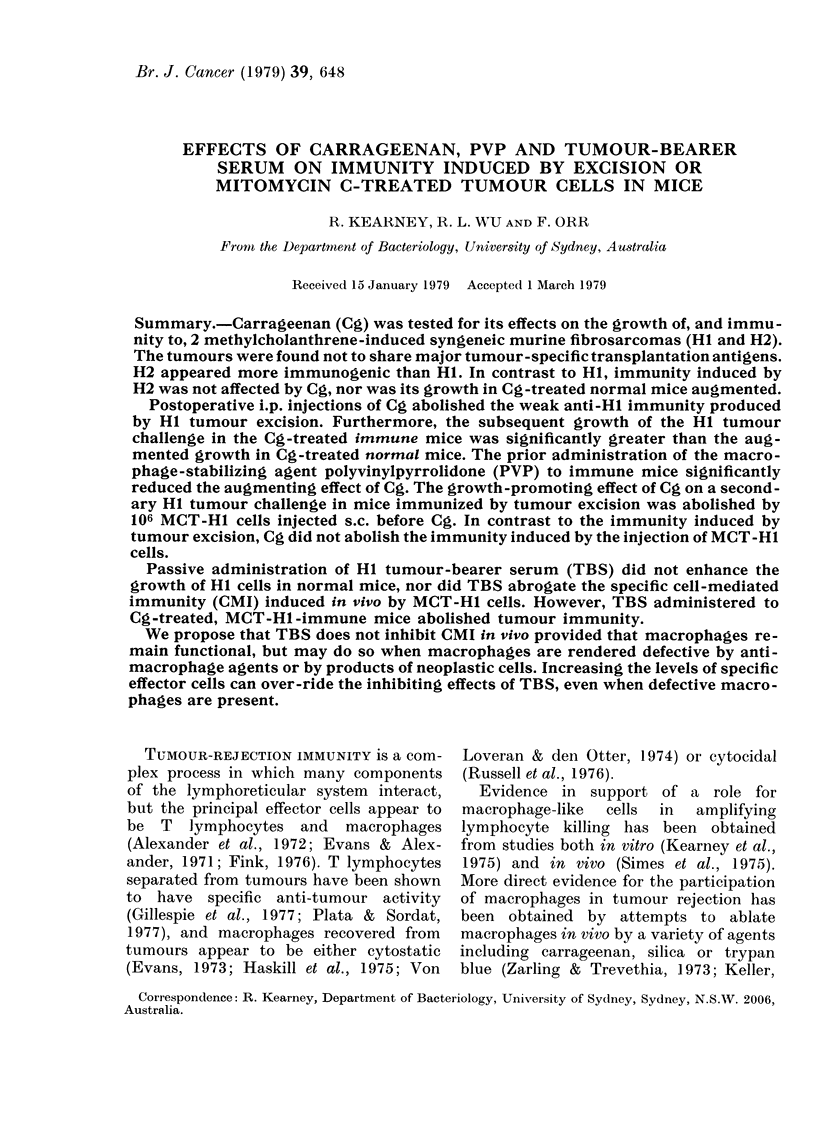

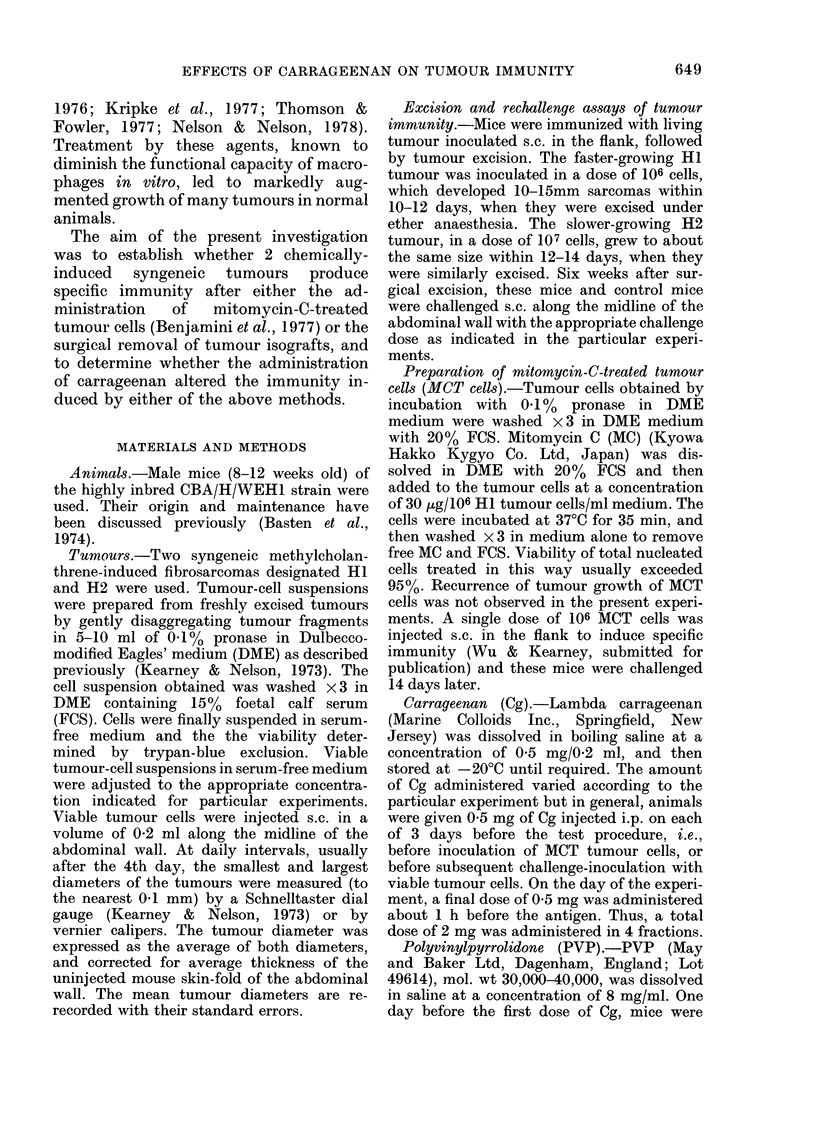

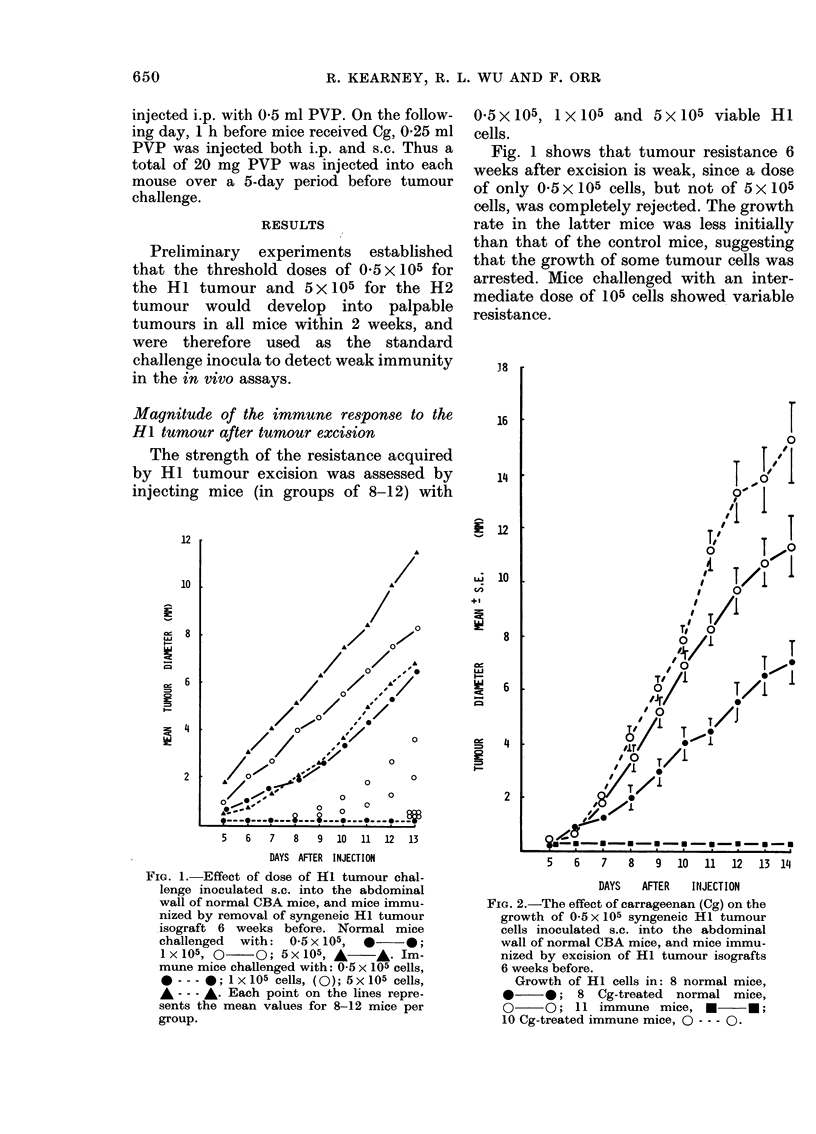

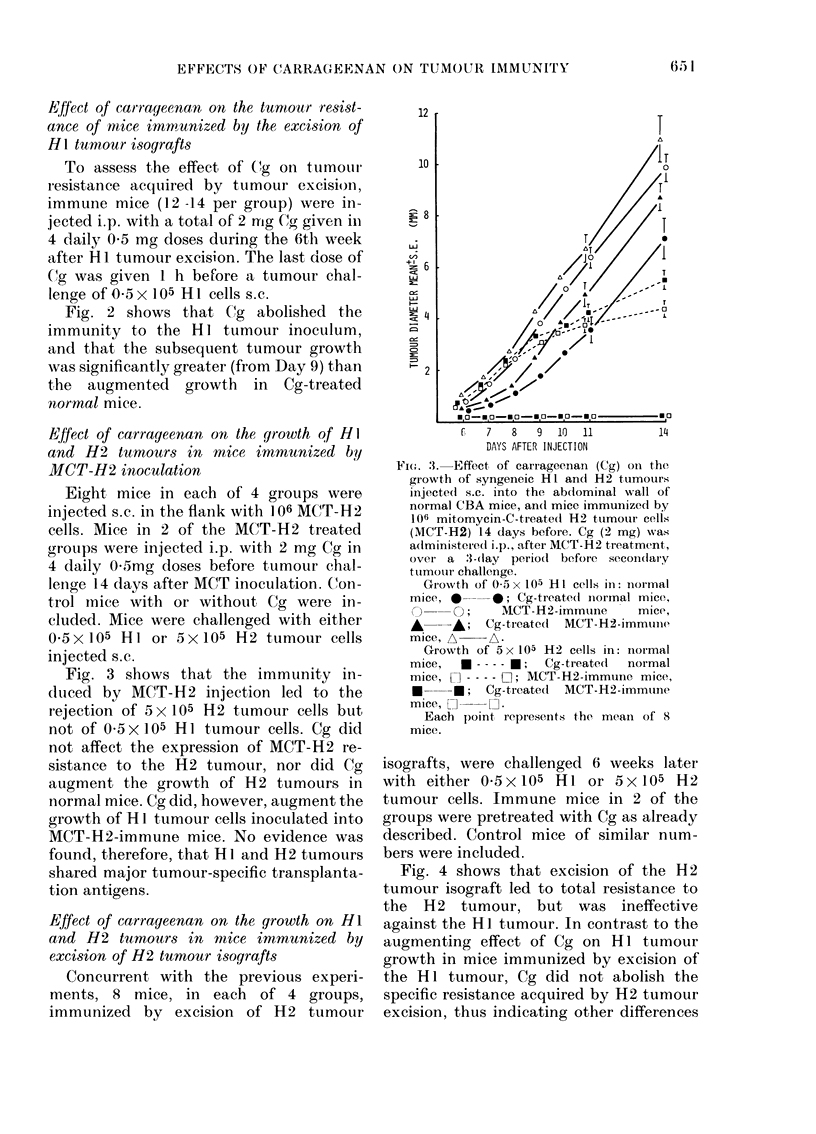

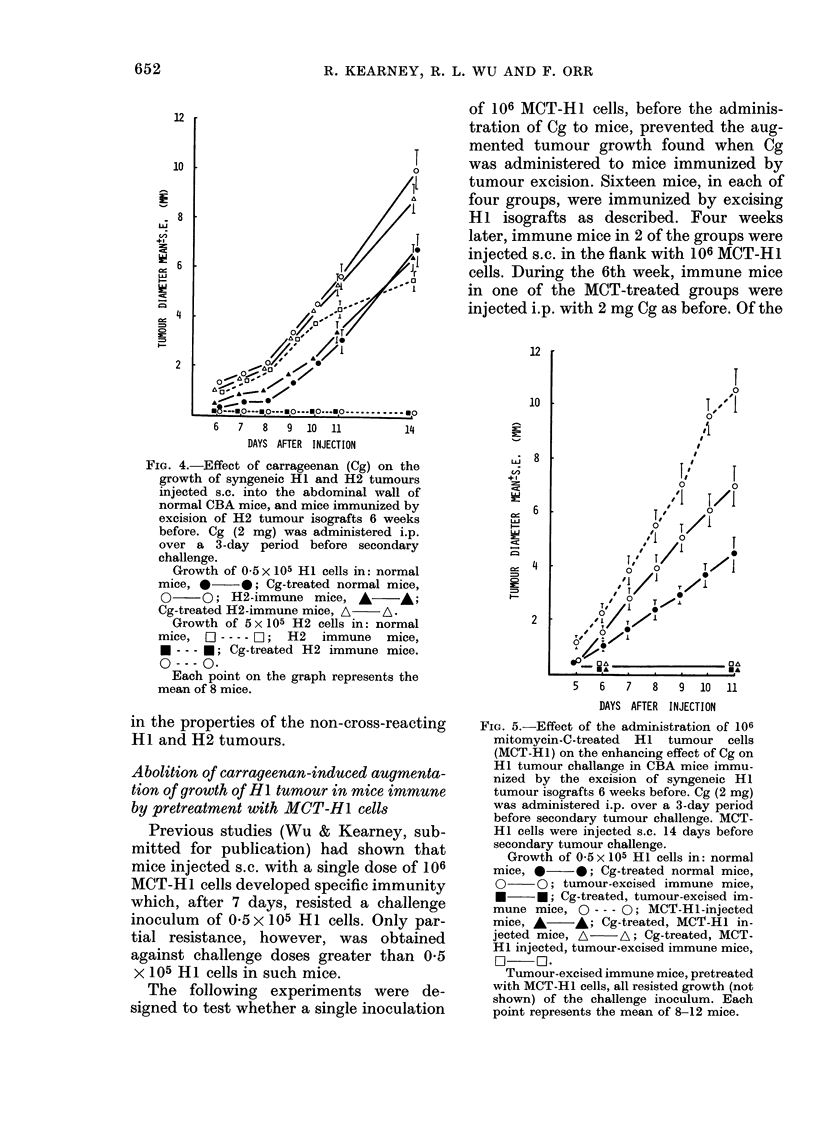

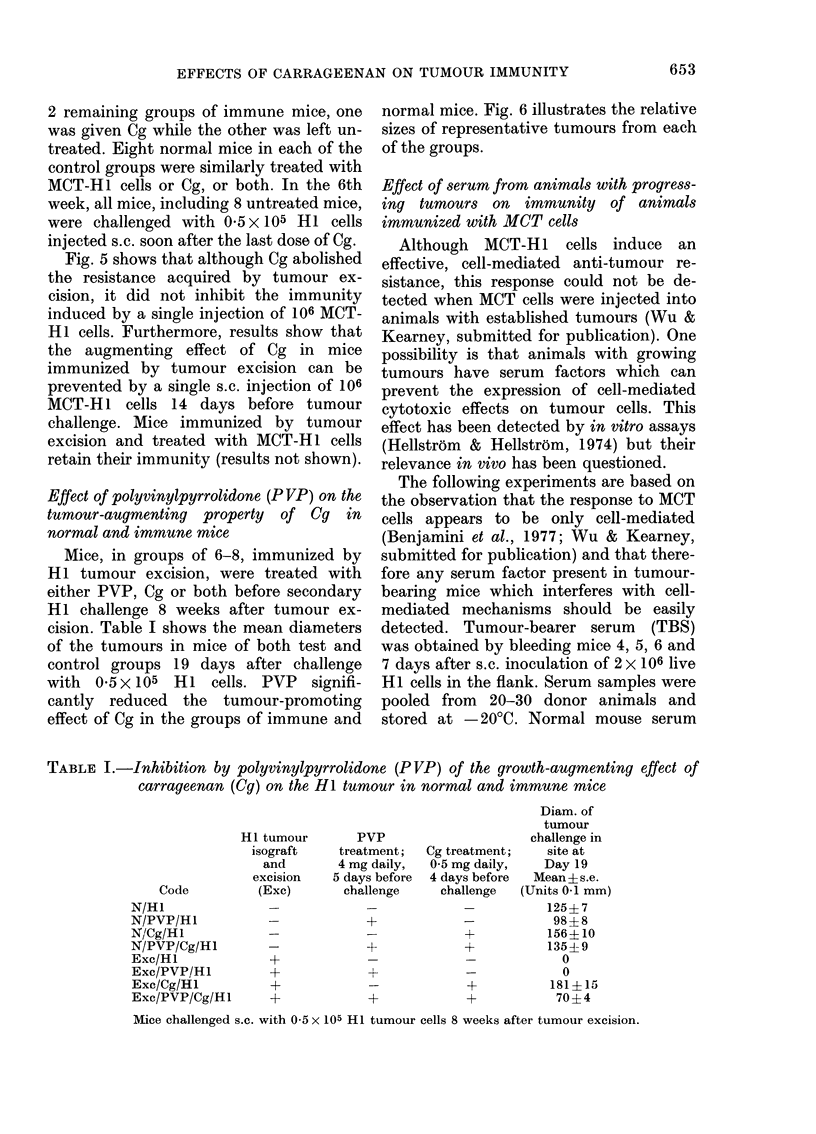

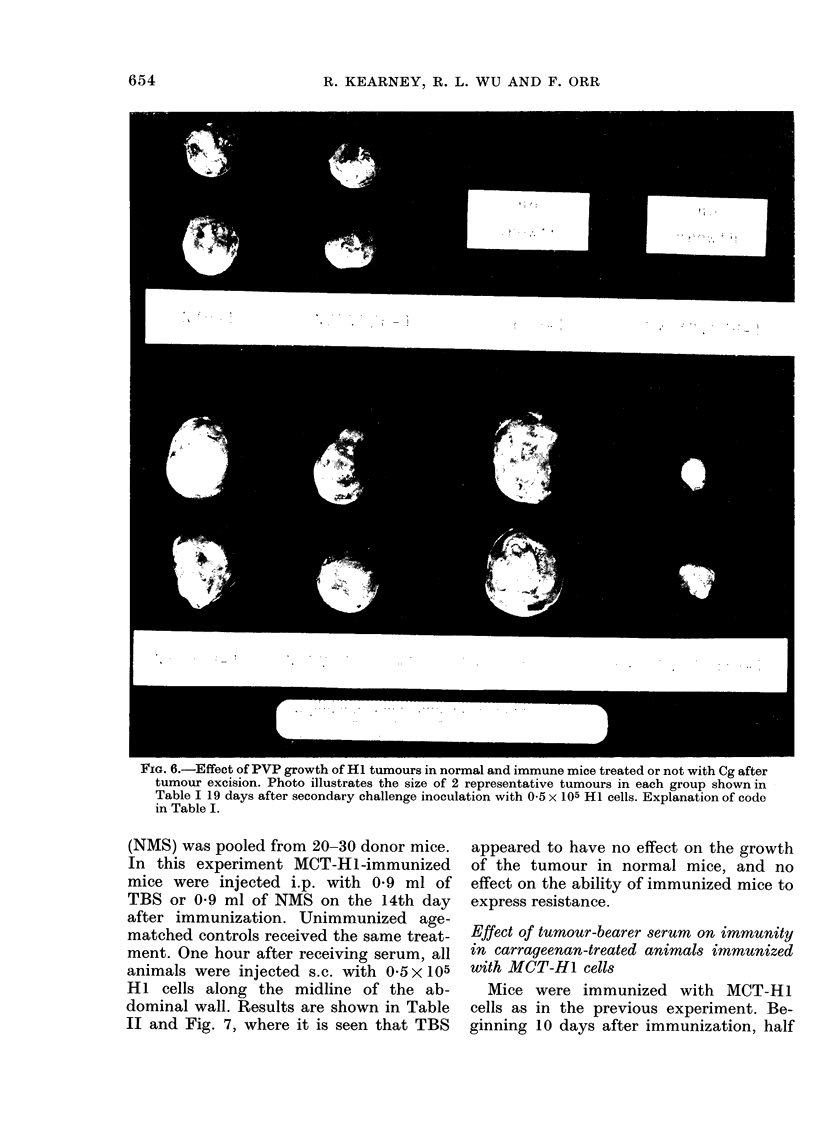

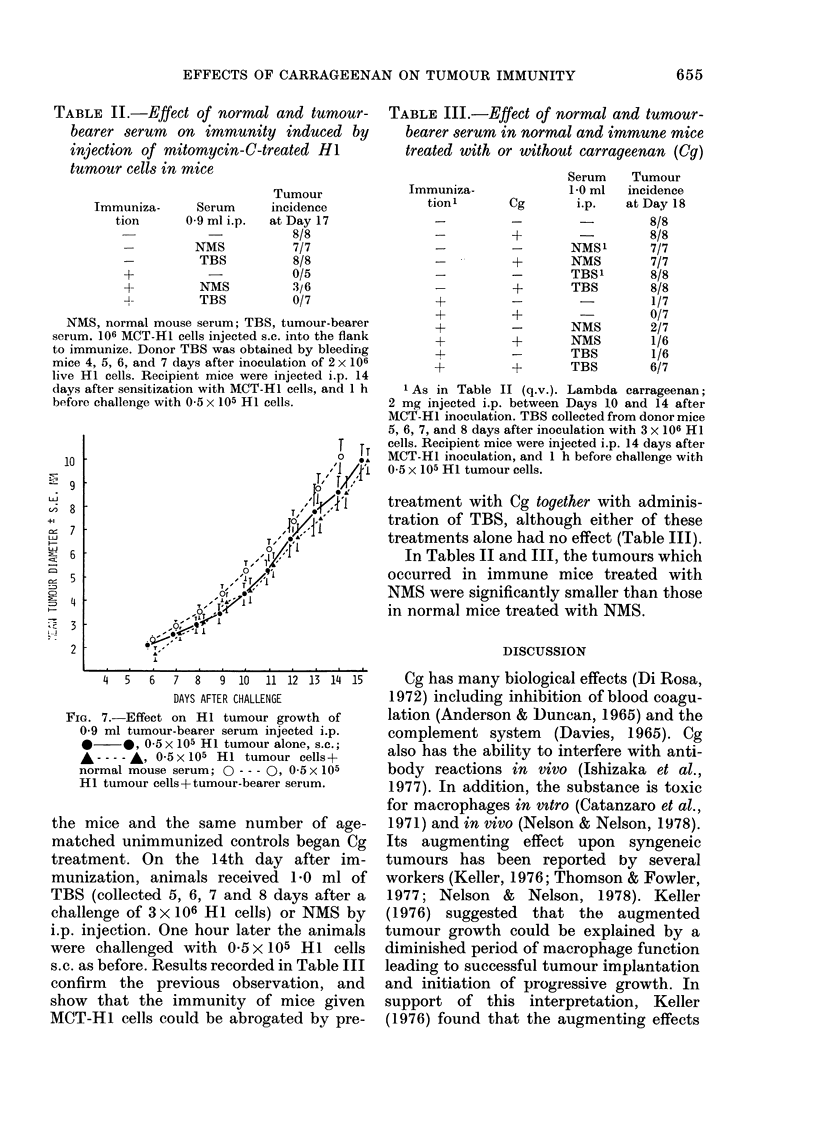

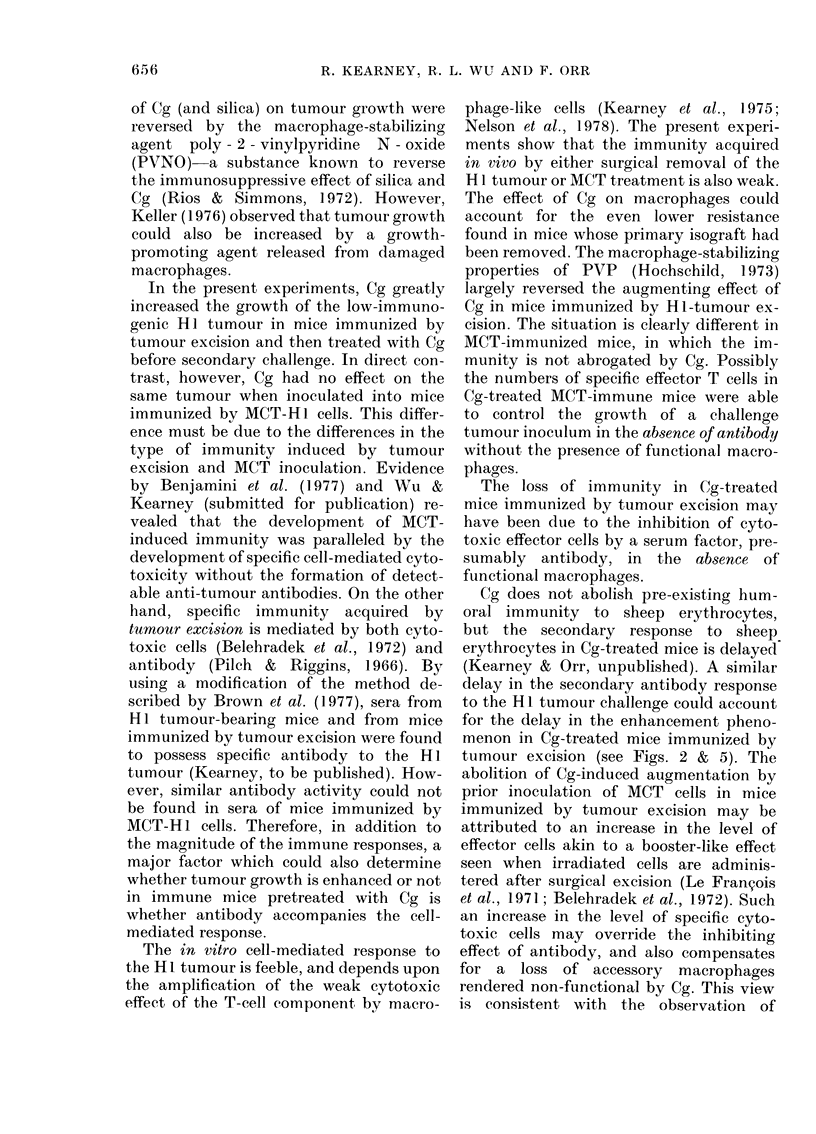

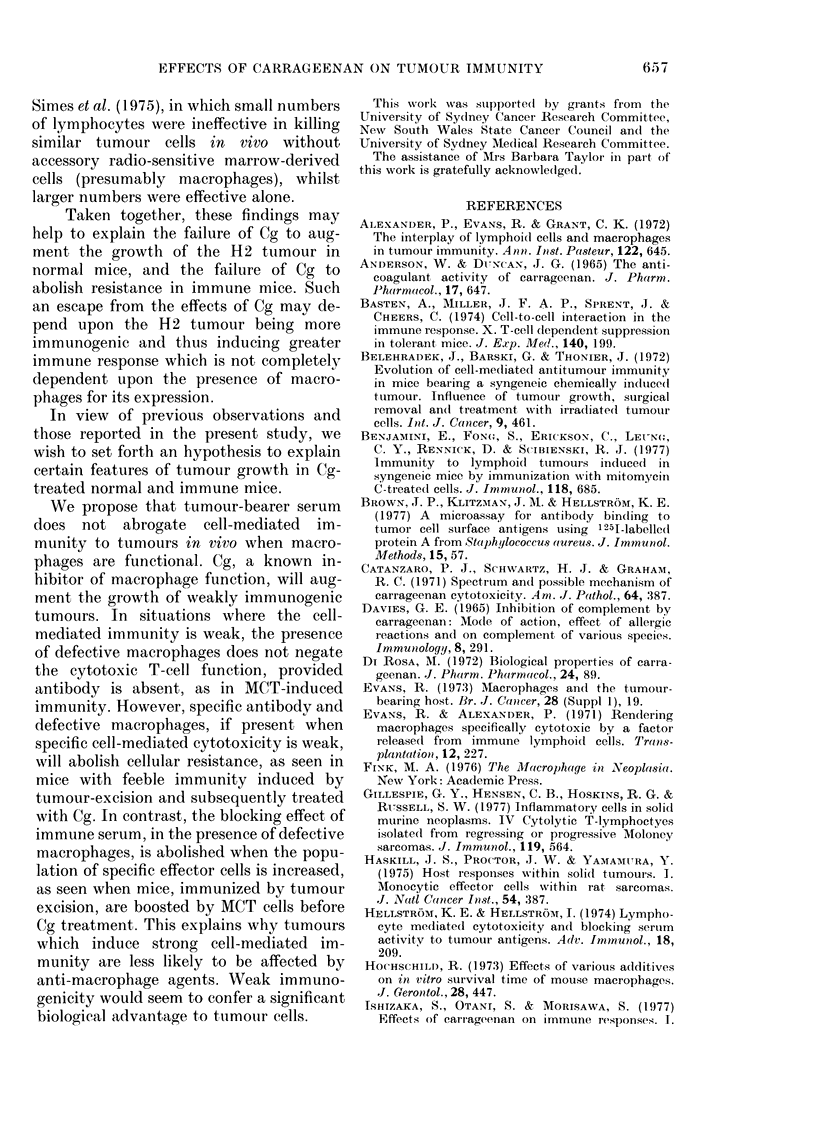

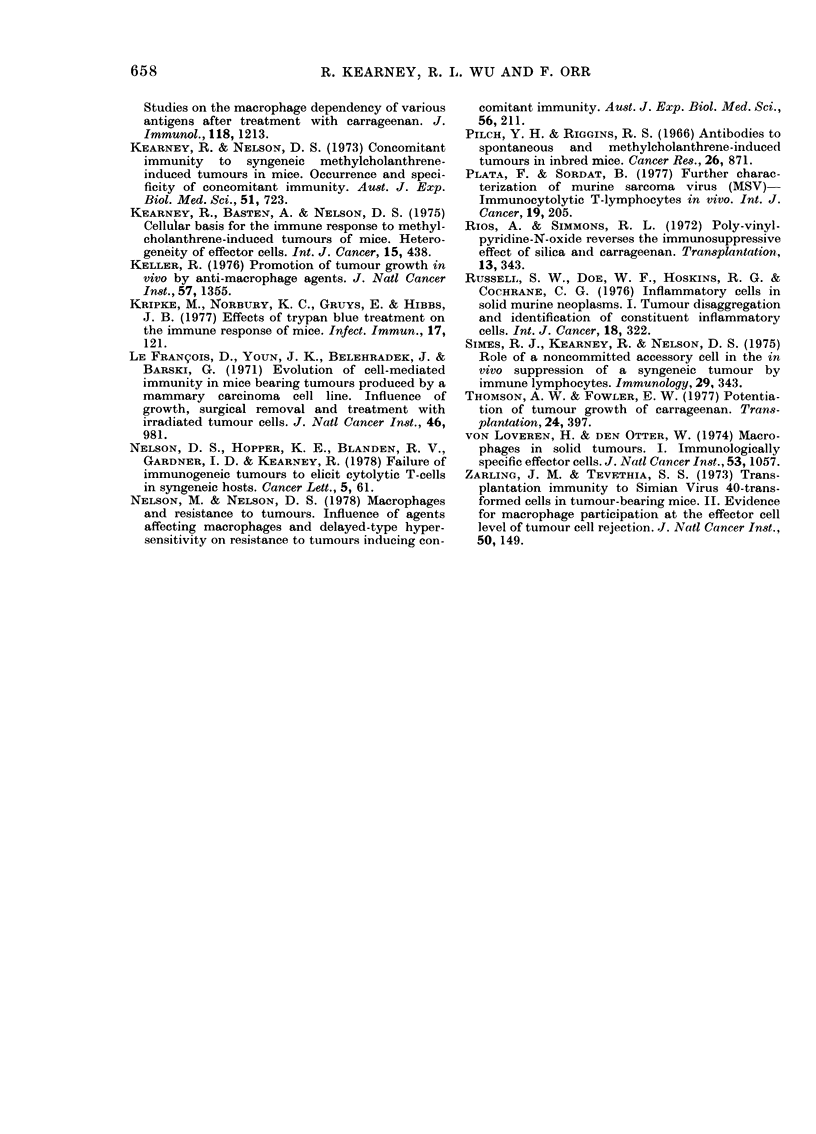


## References

[OCR_01122] Alexander P., Evans R., Grant C. K. (1972). The interplay of lymphoid cells and macrophages in tumour immunity.. Ann Inst Pasteur (Paris).

[OCR_01127] Anderson W., Duncan J. G. (1965). The anticoagulant activity of carrageenan.. J Pharm Pharmacol.

[OCR_01132] Basten A., Miller J. F., Sprent J., Cheers C. (1974). Cell-to-cell interaction in the immune response. X. T-cell-dependent suppression in tolerant mice.. J Exp Med.

[OCR_01138] Belehradek J., Barski G., Thonier M. (1972). Evolution of cell-mediated antitumor immunity in mice bearing a syngeneic chemically induced tumor. Influence of tumor growth, surgical removal and treatment with irradiated tumor cells.. Int J Cancer.

[OCR_01146] Benjamini E., Fong S., Erickson C., Leung C. Y., Rennick D., Scibienski R. J. (1977). Immunity to lymphoid tumors in syngeneic mice by immunization with mitomycin C-treated cells.. J Immunol.

[OCR_01162] Catanzaro P. J., Schwartz H. J., Graham R. C. (1971). Spectrum and possible mechanism of carrageenan cytotoxicity.. Am J Pathol.

[OCR_01166] DAVIES G. E. (1965). INHIBITION OF COMPLEMENT BY CARRAGEENIN: MODE OF ACTION, EFFECT ON ALLERGIC REACTIONS AND ON COMPLEMENT OF VARIOUS SPECIES.. Immunology.

[OCR_01172] Di Rosa M. (1972). Biological properties of carrageenan.. J Pharm Pharmacol.

[OCR_01180] Evans R., Alexander P. (1971). Rendering macrophages specifically cytotoxic by a factor released from immune lymphoid cells.. Transplantation.

[OCR_01176] Evans R. (1973). Macrophages and the tumour bearing host.. Br J Cancer Suppl.

[OCR_01191] Gillespie G. Y., Hansen C. B., Hoskins R. G., Russell S. W. (1977). Inflammatory cells in solid murine neoplasms. IV. Cytolytic T lymphocytes isolated from regressing or progressing Moloney sarcomas.. J Immunol.

[OCR_01198] Haskill J. S., Proctor J. W., Yamamura Y. (1975). Host responses with solid tumors. I. Monocytic effector cells within rat sarcomas.. J Natl Cancer Inst.

[OCR_01210] Hochschild R. (1973). Effects of various additives on in vitro survival time of mouse macrophages.. J Gerontol.

[OCR_01232] Kearney R., Basten A., Nelson D. S. (1975). Cellular basis for the immune response to methylcholanthrene-induced tumors in mice. Heterogeneity of effector cells.. Int J Cancer.

[OCR_01225] Kearney R., Nelson D. S. (1973). Concomitant immunity to syngeneic methylcholanthrene-induced tumours in mice. Occurrence and specificity of concomitant immunity.. Aust J Exp Biol Med Sci.

[OCR_01238] Keller R. (1976). Promotion of tumor growth in vivo by antimacrophage agents.. J Natl Cancer Inst.

[OCR_01243] Kripke M. L., Norbury K. C., Gruys E., Hibbs J. B. (1977). Effects of trypan blue treatment on the immune responses of mice.. Infect Immun.

[OCR_01249] Le François D., Youn J. K., Belehradek J., Barski G. (1971). Evolution of cell-mediated immunity in mice bearing tumors produced by a mammary carcinoma cell line. Influence of tumor growth, surgical removal, and treatment with irradiated tumor cells.. J Natl Cancer Inst.

[OCR_01258] Nelson D. S., Hopper K. E., Blanden R. V., Gardner I. D., Kearney R. (1978). Failure of immunogenic tumors to elicit cytolytic T cells in syngeneic hosts.. Cancer Lett.

[OCR_01264] Nelson M., Nelson D. S. (1978). Macrophages and resistance to tumours: influence of agents affecting macrophages and delayed-type hypersensitivity on resistance to tumours inducing concomitant immunity.. Aust J Exp Biol Med Sci.

[OCR_01273] Pilch Y. H., Riggins R. S. (1966). Antibodies to spontaneous and methylcholanthrene-induced tumors in inbred mice.. Cancer Res.

[OCR_01278] Plata F., Sordat B. (1977). Murine sarcoma virus (MSV)-induced tumors in mice. I. Distribution of MSV-immune cytolytic T lymphocytes in vivo.. Int J Cancer.

[OCR_01284] Rios A., Simmons R. L. (1972). Poly-2-vinylpyridine N-oxide reverses the immunosuppressive effects of silica and carrageenan.. Transplantation.

[OCR_01292] Russell S. W., Doe W. F., Hoskins R. G., Cochrane C. G. (1976). Inflammatory cells in solid murine neoplasms. I. Tumor disaggregation and identification of constituent inflammatory cells.. Int J Cancer.

[OCR_01297] Simes R. J., Kearney R., Nelson D. S. (1975). Role of a non-committed accessory cell in the in vivo suppression of a syngeneic tumour by immune lymphocytes.. Immunology.

[OCR_01303] Thomson A. W., Fowler E. F. (1977). Potentiation of tumour growth by carrageenan.. Transplantation.

[OCR_01308] Van Loveren H., Den Otter W. (1974). Macrophages in solid tumors. I. Immunologically specific effector cells.. J Natl Cancer Inst.

[OCR_01312] Zarling J. M., Tevethia S. S. (1973). Transplantation immunity to simian virus 40-transformed cells in tumor-bearing mice. II. Evidence for macrophage participation at the effector level of tumor cell rejection.. J Natl Cancer Inst.

